# Full Optoelectronic Simulation of Lead-Free Perovskite/Organic Tandem Solar Cells

**DOI:** 10.3390/polym15030784

**Published:** 2023-02-03

**Authors:** Marwa S. Salem, Ahmed Shaker, Mohamed Abouelatta, Ahmed Saeed

**Affiliations:** 1 Department of Computer Engineering, College of Computer Science and Engineering, University of Ha’il, Ha’il 55211, Saudi Arabia; 2 Department of Electrical Communication and Electronics Systems Engineering, Faculty of Engineering, Modern Science and Arts University (MSA), Cairo 12556, Egypt; 3 Engineering Physics and Mathematics Department, Faculty of Engineering, Ain Shams University, Cairo 11517, Egypt; 4 Electronics and Electrical Communications Department, Faculty of Engineering, Ain Shams University, Cairo 11517, Egypt; 5 Electrical Engineering Department, Future University in Egypt, Cairo 11835, Egypt

**Keywords:** lead-free, perovskite, organic, tandem, solar cell, SETFOS

## Abstract

Organic and perovskite semiconductor materials are considered an interesting combination thanks to their similar processing technologies and band gap tunability. Here, we present the design and analysis of perovskite/organic tandem solar cells (TSCs) by using a full optoelectronic simulator (SETFOS). A wide band gap lead-free ASnI_2_Br perovskite top subcell is utilized in conjunction with a narrow band gap DPPEZnP-TBO:PC61BM heterojunction organic bottom subcell to form the tandem configuration. The top and bottom cells were designed according to previous experimental work keeping the same materials and physical parameters. The calibration of the two cells regarding simulation and experimental data shows very good agreement, implying the validation of the simulation process. Accordingly, the two cells are combined to develop a 2T tandem cell. Further, upon optimizing the thickness of the front and rear subcells, a current matching condition is satisfied for which the proposed perovskite/organic TSC achieves an efficiency of 13.32%, *J_sc_* of 13.74 mA/cm^2^, and *V_oc_* of 1.486 V. On the other hand, when optimizing the tandem by utilizing full optoelectronic simulation, the tandem shows a higher efficiency of about 14%, although it achieves a decreased *J_sc_* of 12.27 mA/cm^2^. The study shows that the efficiency can be further improved when concurrently optimizing the various tandem layers by global optimization routines. Furthermore, the impact of defects is demonstrated to highlight other possible routes to improve efficiency. The current simulation study can provide a physical understanding and potential directions for further efficiency improvement for lead-free perovskite/organic TSC.

## 1. Introduction

Solar cells are commonly established as an indispensable means to conquer global pollution issues [1]. Consequently, a broad range of solar cells have been proposed and developed. In this context, crystalline silicon (c-Si) solar cells have dominated the terrestrial global solar market with a market share of over 90% [2], having a record power conversion efficiency (PCE) of more than 26% [3], while their Shockley–Queisser (S-Q) limit is 32% [4]. Many modifications were explored to promote the development of Si-based solar cells in order to attain lower fabrication costs. These include structures with micro-gratings which have vertical sidewall emitter contacts [5], micro-pillars with Cu nanoparticles [6], nanorods with a high-doped pn-junction in the radial path [7], and npn microstructure based on high-doped wafers [8,9,10,11], among other structures [12]. However, other counterparts are being investigated to achieve an optimum efficiency-to-cost ratio. In this regard, many promising materials have been recognized for potential use in thin-film solar cells (TFSCs). These materials reveal growing PCEs, for example, over 25% for perovskite, 21% for CdTe, 23% for copper indium gallium selenide (CIGS), and 19% for organic TFSCs [13,14,15]. Of these materials suitable for TFSCs, perovskite and organic materials have captured the attention of many researchers, owing to their common advantages involving their low cost, low-temperature solution-processing, and simple fabrication steps [16].

Notably, the PCEs of practical perovskite and organic single junction solar cells are nonetheless smaller than the expected theoretical values. A prospective approach to breaking the performance limit of single-junction solar cells is the production of tandem solar cells (TSCs), which involves merging a wide band gap top subcell with a narrow band gap back subcell. Although the PCE of all-perovskite two-terminal (2T) TSCs has progressively advanced from 17.0% to 24.8% in the last few years [17,18], difficulties in producing all-perovskite TSCs are encountered, which come from serious stability issues or non-intentional p-type doping in the bottom cell, which causes considerable degradation of cell performance [19]. As a result, c-Si has been utilized as a back cell combined with a wide band gap perovskite solar cell that acts as a front cell. Although high efficiencies were reported in the literature for perovskite/c-Si TSC [20,21,22], these tandems are not consistent with low-temperature and low-cost manufacture appearing for perovskite solar cell (PSC) technology. In this respect, organic solar cells (OSCs) show a suitable choice as narrow-gap rear cells. Thus, instead of high-cost c-Si cells, OSCs are considered promising and desirable alternatives.

Many efforts have been devoted to exploring perovskite/organic TSCs and some of these tandems showed high efficiencies [23,24,25,26,27]. The first 2T all-inorganic perovskite/organic TSC achieved a PCE of slightly higher than 15% [23]. By utilizing more advanced subcells, the PCE has increased to about 18% [24]. Further, by designing a TSC with CsPbI_2_Br and D18:Y6 (where D18 is a copolymer donor and Y6 newly developed non-fullerene acceptor) acting as a front perovskite cell and a rear organic cell, respectively, a PCE of 20.18% has been recorded [25]. The Cs_0.25_FA_0.75_Pb(I_0.6_Br_0.4_)_3_ perovskite was used as the front subcell (having an energy gap of 1.79 eV) along with a ternary system of PM6 (a D–π–A type copolymer), Y6, and P_71_CBM as a bottom subcell (having an energy gap of 1.36 eV). After fabricating the TSC based on the mentioned absorbers, a certified PCE of 22.95% has been recorded [26]. Moreover, a TSC with a stabilized PCE of 24% has been attained with FA_0.8_Cs_0.2_Pb(I_0.5_Br_0.5_)_3_ and the binary bulk heterojunction PM6 and Y6 as the top and bottom photoactive absorbers, respectively [27]. Despite the diversity of these efforts, the PCEs and the performance of perovskite/organic TSCs still lag behind those of other tandem configuration TFSC candidates, implying that more work is necessary to boost the performance of the perovskite/organic TSCs.

Apart from this, simulation studies can help scientists and researchers to reasonably select materials based on their available experimental data from the literature [28,29]. Furthermore, by utilizing device simulators, one can optimally design and thereby predict the performance of several types of solar cells [30]. In this context, some simulation-based efforts, using different simulators, have been conducted regarding TSCs. A significant amount of all-perovskite, either lead-based, lead-free, or low lead content TSC structures, has been numerically presented, giving possible paths for efficiency-boosting [31,32,33,34]; however, very few studies could be found in the literature regarding perovskite/organic TSC simulation. In [35], the authors demonstrated a theoretical study for modeling various hybrid perovskite/organic TSCs, revealing a maximum PCE of 19.81% for the system: CsPbI_3_/PDPPSDTPS:PCBM. The simulation of other multi-junction configurations was also presented [35]. Moreover, D. Rossi et al. provided electro-optical simulations for the design of semitransparent perovskite/organic TSCs. The photoactive absorber materials used in this study were MAPbBr_3−*x*_ Cl _*x*_ and PTB7-Th:IEICO-4F, resulting in a PCE of about 15% [36]. In all these mentioned studies, lead-based perovskites were utilized.

It can be observed from the previous discussion that there are no efforts to date, either experimentally or by using simulation tools, to investigate the lead-free/organic TSCs. Utilizing lead-free PSCs is crucial in order to alleviate the issues encountered in toxic lead-based cells for broader commercialization. Thus, lead-free perovskites should be researched in conjunction with organic materials as promising contenders. However, wide band gap lead-free perovskite materials are rarely investigated [37]. Furthermore, as the efficiency achieved from using such materials is still low compared to lead-based solar cells, there is a significant need to develop lead-free PSCs combined with OSCs in tandem configurations for future PV applications. Remarkably, simulation can provide a physical understanding and potential directions for further efficiency improvement for such type of TSC. Therefore, in this paper, we investigate the combination of a wide band gap (1.61 eV) ASnI_2_Br (GA_0.06_(FA_0.8_Cs_0.2_)_0.94_SnI_2_Br) lead-free PSC with a narrow band gap (1.33 eV) DPPEZnP-TBO:PC61BM in a 2T tandem configuration. The used cells are based on previously published experimental PSC and OSC architectures [37,38]. The main target in choosing this promising perovskite lead-free material in our simulation is to achieve a non-toxic tandem solar cell. The issue related to the oxidation of Sn^2+^ into Sn^4+^ in Sn-based perovskites forming intrinsic Sn vacancies resulting in an unwanted p-type self-doping was alleviated in the presented ASnI_2_Br perovskite solar cell by the addition of Germanium Iodide (GeI_2_), which led to suppression in Sn vacancies. Furthermore, the fabricated cell incorporates the addition of ethylenediamine dibromide (EDABr_2_), and outstanding enhancements in the optoelectronic properties were observed, resulting in a higher overall solar cell performance and stability. The modifications of GeI_2_ doping, incorporation of EDABr_2_ at A site, EDA passivation resulted in increasing the carrier lifetime of the absorber from 1.1 ns to 22.8 ns, and a balanced charge transfer on the optimized perovskite surface film. As a result, the efficiency was improved from 4.86% to 7.50% which is the best efficiency for wide bandgap lead-free SCs to date [37]. Regarding the organic solar cell chosen, it involves the use of polymer-based HTL and ETL, namely PEDOT:PSS which is an intrinsically conductive polymer, and PFN, which is a conjugated polyelectrolyte semiconducting polymer [38]. This work provides a proof of concept, for the first time, that tin-halide PSCs have the potential to be used as a top subcell in lead-free perovskite/organic TSCs.

## 2. Simulation Methodology and Device Structures

We performed the device analyses by utilizing SETFOS commercial software, which is a device simulator used in the numerical simulation of multilayer solar cells. It is a powerful tool for simulating the optical and electrical properties of perovskite and organic devices. SETFOS helps to direct experimental tests and understand the physics of optical materials as well as optimize the technological parameters of a solar cell structure for better cell performance. The simulator has been widely used and validated for both PSCs [39,40,41] and OSCs [42,43,44].

### 2.1. Simulation Methodology

SETFOS software package is based on mathematical models to concurrently simulate the optical and electrical properties of the PV cell where the modeling steps are incorporated [45]. First, regarding optical simulation, SETFOS uses the transfer matrix method (TMM) to evaluate the optical properties of multilayer structures [46]. For each wavelength, the absorbance of each thickness is given by

(1) A(λ)=1−R(λ)−T(λ)

The expression of the transmittance is given by

(2) T=exp(αd)

where *d* is the layer thickness. The passage of light results in an attenuation according to the coefficient *α*(*λ*), defined by

(3) $$\alpha =\frac{4\pi k}{\lambda }$$

where *k* represents the extinction parameter. Next, the simulation continues to the electrical part as follows. The light generates excitons which will dissociate to form free electrons and holes. The charge transport phenomenon is described by the semiconductor continuity equations of electrons and holes, as represented in the following equations:

(4) $$\frac{dn}{dt}=\frac{\vec{\nabla }.\vec{J_{n}}}{-q}-R_{Langevin}-R_{nt}+g_{np}G_{n}$$

(5) $$\frac{dp}{dt}=\frac{\vec{\nabla }.\vec{J_{p}}}{q}-R_{Langevin}-R_{pt}+g_{np}G_{p}$$

where *n* and *p* denote the density of electrons and holes, while *R_nt_* and *R_pt_* describe the recombination rates due to electrons and holes, respectively. The last terms in the above equations describe optical charge generation where *g_np_* is the generation efficiency. These equations describe charge species that are coupled with Poisson’s equation,

(6) $$\vec{\nabla }.\left(\varepsilon \varepsilon_{o}\vec{E}\right)=q\left(p-n+p_{t}-n_{t}-A_{Doping}+D_{Doping}\right)$$

Here, *ε* is the dielectric constant, and *E* is the electric field. The doping terms *A_Doping_* and *D_Doping_* represent acceptor and donor concentrations. Langevin recombination *R_Langevin_*, appearing in Equation (4), occurs in organic semiconductors and it is a bimolecular process with the rate,

(7) $$R_{Langevin}=\eta \left(\mu_{n}+\mu_{p}\right)\frac{q}{\varepsilon }\left(np-n_{i}^{2}\right)$$

which is a function of the local charge concentrations and the electron and hole mobilities (*μ_n_* and *μ_p_*) [47]. *n_i_* is the intrinsic concentration at thermal equilibrium while *η* signifies the Langevin recombination efficiency. Trapping and de-trapping of carrier traps are defined by the trap rate equation. For instance, the electron trap can swap electrons or holes with the LUMO (Lowest Unoccupied Molecular Orbital) level at the rate *R_te_* or with the HOMO (Highest Occupied Molecular Orbital) level at the rate *R_th_* [48], respectively,

(8) $$\frac{dn_{t}}{dt}=R_{te}-R_{th}$$

Equation (8) describes Shockley–Read–Hall (SRH) recombination where free electrons are captured in the trap and then recombination with a free hole occurs.

A crucial aspect that allows accurate electrical simulations of tandem cells within SETFOS framework is the hopping interface model [49] which can efficiently describe the charge transport between the subcells of monolithic 2T tandem cells. This model is built on the basis of the theory of thermally activated hopping [50]. Both intra- and inter-band transitions are handled by calculating the corresponding rates for the charge transfer [51]. Further details about the simulation equations and the numerical techniques are found in Ref. [45].

### 2.2. Device Structures and Material Parameters

**Figure 1:**
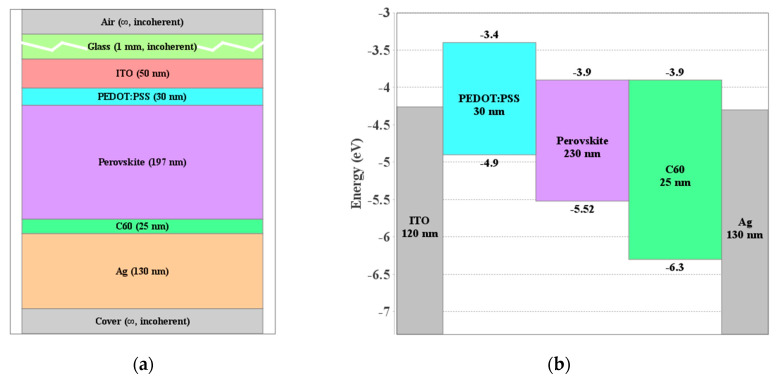
(**a**) Layer stack representing the perovskite cell structure and (**b**) energy band alignment (before contact) of various layers in the PSC.

Next, we present the device structures of the PSC and OSC utilized in designing the TSC. Our work is based on experimental solar cells, a wide band gap lead-free PSC, and a narrow band gap OSC. The PSC inverted structure is FTO/PEDOT:PSS/perovskite/C60/BCP/Ag where the perovskite material is GA_0.06_(FA_0.8_Cs_0.2_)_0.94_SnI_2_Br with a band gap of 1.62 eV [37]. This wide band gap perovskite is treated by doping of GeI_2_ and incorporation of EDABr_2_ at A site [37]. A schematic representation of the PSC configuration is displayed in Figure 1a showing the main stacked layers produced within SETFOS environment. The primary material parameters of the various layers are addressed in Table S1 in the Supplementary Materials. The LUMO and HOMO levels are calculated based on measurements of the valence band level [37]. A reported PCE of 7.50% was addressed [37]. This cell will be used as the top cell. Thin layers of PEDOT:PSS and C60 are applied as hole and electron transport layers, respectively. The front and back electrodes are created by ITO and Ag, respectively. 

**Figure 2:**
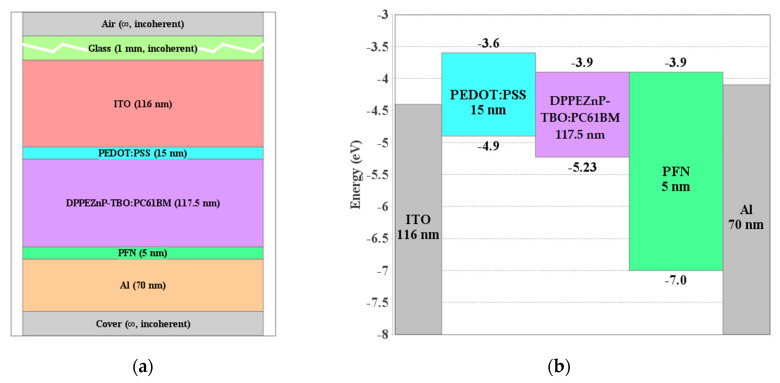
(**a**) Layer stack representing the organic cell structure and (**b**) energy band alignment (before contact) of various layers in the OSC.

Regarding the active layer of the chosen OSC, DPPEZnP-TBO:PC_61_BM is utilized. The molecular structures of the DPPEZnP-TBO and fullerene PC_61_BM can be found in Figure S1 in the Supplementary Materials. A description of the PSC is shown in Figure 2a showing the main stacked layers created by SETFOS, while the primary material parameters of the various layers are recorded in Table S2 in the Supplementary Materials. This organic cell will be utilized as the rear cell in the tandem configuration. Thin films of PEDOT:PSS and PFN are employed as hole and electron transport layers, respectively. The front and back contacts are formed by ITO and Al, respectively.

The HOMO and LUMO levels were taken from [37,38]. Regarding C60, the electrical parameters are taken from Ref. [52], while they are taken from Ref. [51] for PEDOT. The values of defect density, trap energy position, and electron and hole capture rates are obtained from the fit versus the experimental illuminated *J-V* characteristics as will be seen hereafter.

### 2.3. Sub-Cells Calibration

**Figure 3:**
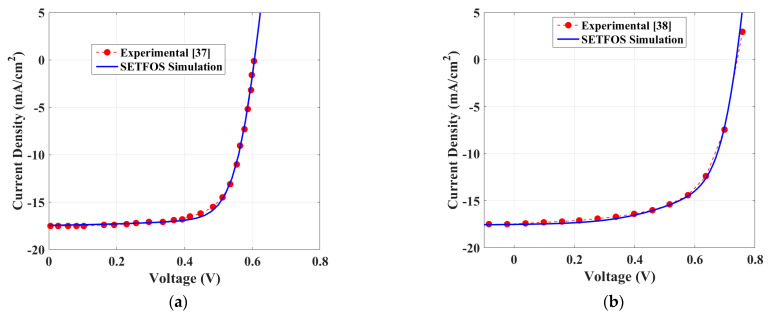
Illuminated *J-V* characteristics obtained by fitting of the experimental with the SETFOSS model: (**a**) PSC and (**b**) OSC.

Now, a SETFOS simulation deck is presented for both perovskite and organic solar cells that is calibrated to experimental data of reported device structures where the incident light illumination used is AM1.5G. The calibration is accomplished by fitting the measured current density-voltage (*J-V*) curves versus those acquired from coupled opto-electrical simulations. This calibration process is achieved by applying the values of material and technological parameters that have been extracted from the experimental device data or obtained from the literature as discussed herein. In addition, the optical parameters, such as the refractive index and extinction coefficient, of the distinct OSC and PSC layers, were also collected from the literature [37,53,54,55]. More details about the optical parameters data can be found in Table S3 in the Supplementary Materials. The recombination parameters are adjusted utilizing global multi-parameter optimization sequences to fit the experimental *J-V* data. The results, displayed in Figure 3a for the PSC and in Figure 3b for the OSC, show a satisfactory agreement between the calibration of the *J-V* data versus measurements indicating the confirmation of the material parameters and physical models implemented in SETFOS TCAD simulator. Further, the quantitative results of the experimental and simulated PV parameters (including short-circuit current (*J_sc_*), open-circuit voltage (*V_oc_*), fill factor (FF), and power conversion efficiency (PCE)) are reported in Table 1.

## 3. Results and Discussions

In this section, we provide the tandem implementation and the corresponding simulation results. Firstly, the tandem cell is presented in which the PSC and OSC, whose parameters are provided in the last section, via a hopping interface. In all simulations, the interface is assumed to be nearly ideal. It should be pointed out here that, in order to make a 2T TSC, the interconnect between the front and rear subcells should be Ohmic. This can be rendered by thin layers in the order of 1 nm of metal (like Ag or Au) [16,56,57]. Additionally, the interconnection can be utilized by InO layer which was tested experimentally and proved both advantageous electrical and optical properties as it behaves like a metal besides its low optical and electrical losses [27].

### 3.1. Tandem Configuration

**Figure 4:**
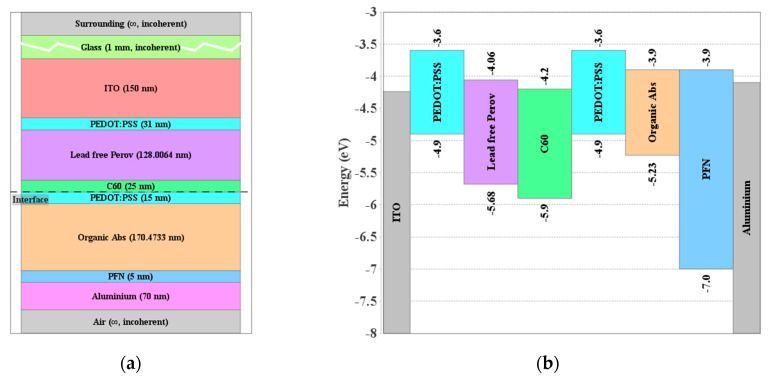
(**a**) Layer stack for the proposed perovskite/organic tandem cell and (**b**) energy band alignment (before contact) of materials in the cell stack.

The proposed TSC is composed of the experimentally based PSC and OSC as discussed herein. A schematic representation of the tandem configuration is exhibited in Figure 4a. The energy alignment before contact is also demonstrated in Figure 4b. The initial layer thicknesses are taken as the reported experimental values mentioned in the previous section. The ITO front contact is assumed to have a work function of about 4.3 eV while the carrier injection is modeled through thermionic boundary conditions. Regarding the Al back contact, it is supposed to be ohmic having a work function of 4.1 eV.

### 3.2. Optical versus Optoelectronics Optimization

**Figure 5:**
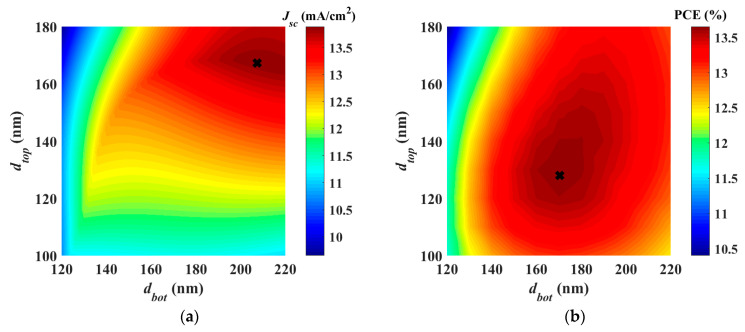
(**a**) Limiting photocurrent variation vs. the thickness of top (*t_top_*) and bottom (*t_bot_*) absorbers based on optical simulation. (**b**) Power conversion efficiency variation vs. the thickness of front and rear absorbers based on full coupled optoelectronic simulation. The black crosses point to the result of the direct global optimization.

By employing the listed material parameters, we first perform absorber thickness optimization through optical simulation only under normal AM1.5G illumination. In this simulation, the thickness of both top and bottom absorbers is varied (while keeping other parameters unchanged), and the limiting photocurrent is extracted as displayed in Figure 5a. A black cross is also shown in the figure which represents the result of the global optimization for the thicknesses. The optimization target here is that the minimum of the subcell currents is maximized. This means that maximum performance is achieved at the condition of current matching. The top and bottom thickness, according to this double sweep or optimization, is *d_top_* = 167.284 nm and *d_bot_* = 207.407 nm, respectively. This gives the current matching situation resulting in a short circuit current density of 13.886 mA/cm^2^.

Next, full optoelectronic optimization is accomplished by searching for the maximum output power conversion efficiency of the tandem. The result of the direct global optimization routine is shown as a black cross in Figure 5b which reveals the variation of the efficiency regarding the variation of absorber thicknesses. The thickness of the front and bottom subcells, according to this optoelectronics optimization, is *d_top_* = 128.006 nm and *d_bot_* = 170.473 nm, respectively. The achieved maximum efficiency is 13.99%. It should be pointed out here that the optimization takes much less time than the conventional sweep.

**Figure 6:**
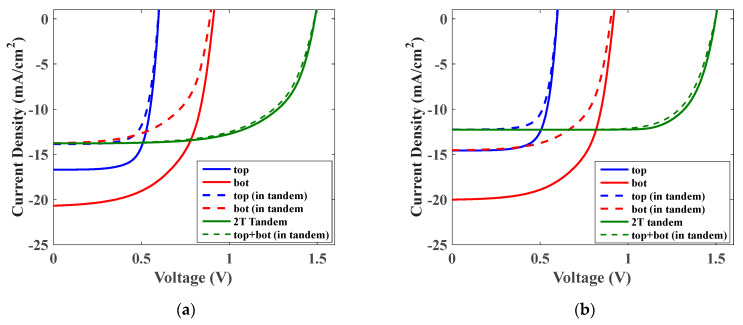
Illuminated *J-V* characteristics based on tandem parameters extracted from (**a**) optical simulation and (**b**) full optoelectronic simulation.

Based on the optimum values of the thicknesses of the absorbers, the *J-V* curves of the optical and optoelectronic optimizations are plotted as displayed in Figure 6a,b, respectively. The figures display the illuminated characteristics of the individual single junction cells, the filtered subcells and the comparison of the full tandem characteristics with the sum of the filtered subcell curves (which assumes a perfect loss-free interconnect). Further, the major performance metrics of the tandems are listed in Table 2. Regarding optical optimization, one can observe a situation with a greater short circuit current, but the electrical performance is worse. On the other hand, although the full opto-electrical optimization gives a lower *J_sc_*, it achieves higher efficiency. The reason is that the non-radiative losses (which are absent in the optics-only model) increase strongly with the thickness of the absorber layers (which will be larger in the optical optimization because of larger generation), especially in the case of low mobility materials such as the organic and lead-free perovskite absorbers. The evolution of the maximum efficiency obtained from the full optoelectronic model illustrates the superiority of the approach to obtain device efficiencies substantially greater than those found by optical optimization only.

**Figure 7:**
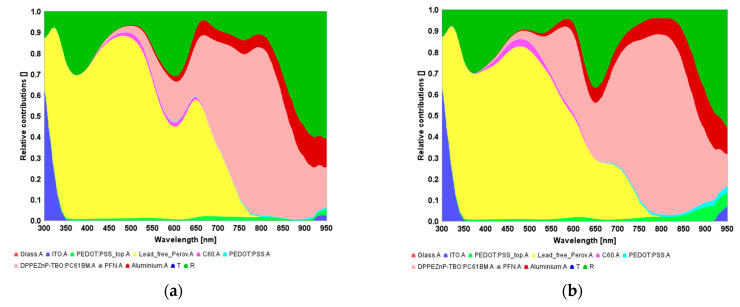
Resolved absorptance spectra as obtained from (**a**) optical simulation and (**b**) coupled simulations.

**Figure 8:**
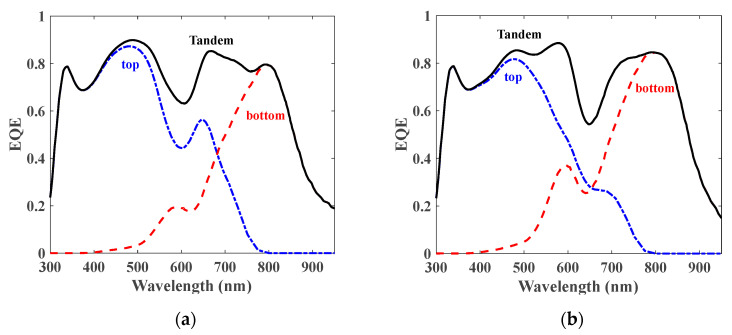
EQE characteristics of sub-cells and tandem cells based on (**a**) optical simulation and (**b**) coupled opto-electrical simulations.

To offer a physical perception of the optical behavior of the tandem cell regarding the optical and optoelectronic optimization techniques, the wavelength-dependent reflectance and absorbance spectra of the various layers are plotted as seen in Figure 7. The useful absorption areas in lead-free perovskite and organic layers are shown by yellow and pink colors, respectively. The green zone signifies the reflection loss, while the other colored regions characterize the absorption losses in the distinctive layers other than the absorbers (such as ETLs, HTLs, and contacts). It should be pointed out that the integration of the absorber spectra zones gives the subcell photocurrents which are the same for the case of optical optimization, whereas they are different in the two subcells of the tandem for the case of the coupled simulation. Moreover, the EQE characteristics of the two sub-cells and tandem cells are presented in Figure 8 based on optical simulation (see Figure 8a) and coupled opto-electrical simulations (see Figure 8b).

### 3.3. Tandem Characteristics under Full Optoelectronic Optimization

**Figure 9:**
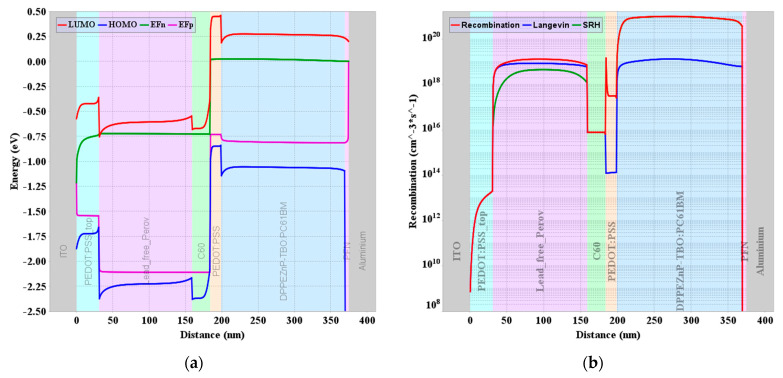
(**a**) Energy band profile at MPP showing the alignment of electron and hole QFLs (*E_Fp_* and *E_Fn_*). (**b**) Recombination rate along the tandem cell at MPP.

In this subsection, we proceed with the design of the perovskite/organic tandem cell by using the parameters obtained from the full optoelectronic optimization. Firstly, in order to highlight the physics beyond the behavior of the tandem, we draw the energy band diagram at the maximum power point (MPP) operation as exhibited in Figure 9. It can be depicted in the figure that there is an alignment of the quasi-Fermi level (QFL) at the interface. This is translated to a nearly zero voltage drop at the interface which reveals an efficient operation of the recombination junction that is modeled through the hopping interface model. Moreover, the recombination rate is plotted at MPP and is shown in Figure 9b. The figure illustrates the different types of recombination (SRH and Langevin) along with the total recombination through the whole tandem.

**Figure 10:**
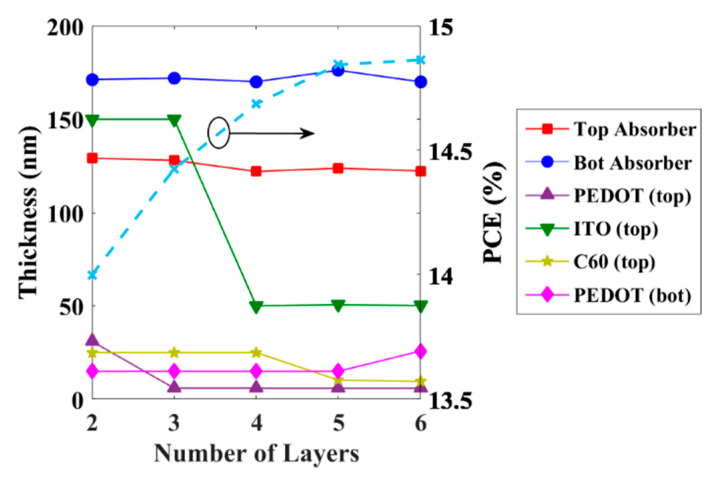
Evaluation of PCE upon layer thickness optimization vs. the number of layers taken in global optimization routines.

For the following simulation, we investigate the optimization of the thicknesses of several tandem layers based on direct global optimization. This is performed by successively subjecting the thicknesses of PEDOT (of the top PSC), ITO, C60, and PEDOT (of the bottom OSC) layers to the optimization routine. The progress of the maximum PCE in accordance with the increase in the number of layers, in this optimization technique, is exhibited in Figure 10. The initial parameters are taken from the double sweep shown in Figure 5b. It can be observed that there is an enhancement in the efficiency when the number of layers encountered in the optimization increases; however, this increase in the PCE is not significant as the maximum obtainable PCE is 14.86% which just exceeds about 0.86% from the initial PCE achieved for two-layer optimization (which is nearly 14%).

**Figure 11:**
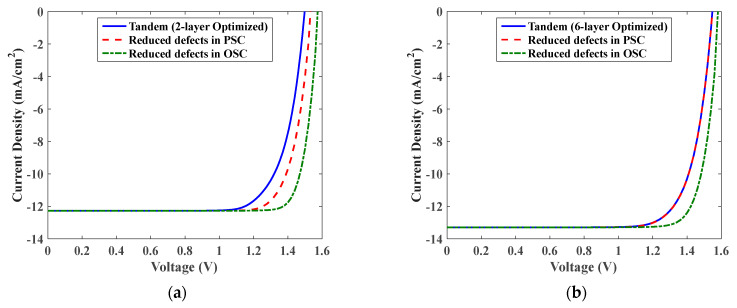
The illuminated *J-V* curves when modifying the defects in PSC and OSC for (**a**) 2-layer optimization and (**b**) 6-layer optimization.

Based on the previous simulations, it is apparent that the structural optimization of this tandem is not the key factor to boost efficiency. The main issue in this tandem is the high density of defects that prevent the cell to reach high performance in addition to the proper design of the conduction band offset (CBO) and valence band offset (VBO). In order to examine the effect of defects, we perform a simulation study in which the defect density is reduced to highlight its influence on the tandem performance. Here, two cases are concerned; the first is when using the parameters obtained in the 2-layer optimization, while the second is when regarding the 6-layer optimization. In each case, we compare three simulation studies. The first study is when using the initial values of defects without modification. The second and third study is when reducing the impact of the defects in the PSC and OSC, respectively. The effect of defects is reduced by decreasing the trap densities to 10% of their initial values listed in Tables S1 and S2. The results are demonstrated in Figure 11a,b for the 2-layer and 6-layer optoelectronic optimization, respectively. The PV parameters of the various case studies are listed in Table 3. As can be inferred from the behavior shown in Figure 11 and the quantitative results presented in Table 3, the impact of PSC defects is much less pronounced than the OSC defects. This trend is more obvious in the case of 6-layer optimization.

Remarkably, further enhancements in device performance by considering various suitable organic or inorganic materials to act as ETLs and HTLs to obtain optimum CBOs and VBOs can be performed to bring this tandem closer to realistic processing.

## 4. Conclusions

In the current work, by combining a wide band gap PSC and a narrow band gap OSC, we have demonstrated a proof of concept of a 2T lead-free perovskite/organic TSC. The TSC, proposed in this study, is based on utilizing a wide band gap lead-free ASnI_2_Br perovskite as a top subcell in combination with a narrow band gap DPPEZnP-TBO:PC61BM heterojunction as an organic bottom subcell. The top and bottom cells were designed according to experimental studies, retaining the same materials and physical parameters. The incorporation of both coherent and incoherent optics along with drift-diffusion besides the appropriate modeling of carrier hopping at the recombination interface enable the optimization of the lead-free perovskite/organic TSC. These simulation features support the full optoelectronic design of TSCs for realistic operation. Upon designing the tandem, we performed two optimization routines, namely, optical-only and full opto-electrical optimization. It was demonstrated that the full optimization gives a higher performance although it does not meet the condition of current matching. The TSC achieves a PCE of 13.99% and 13.32% for full and optical optimization, respectively.

Further, we considered the optimization of multilayers to boost PCE. Upon concurrently globally optimizing six different layers, the TSC achieves an efficiency of less than 15%. Additionally, the impact of the defects in the PSC and OSC has been investigated and it was found that the defects in the bottom cell have a more pronounced impact on the performance than the defects in the top cell. An efficiency of slightly less than 17.5% has been obtained for reduced defects and 6-layer optimization. The detailed analysis, carried out in this simulation study, of the TSC design would open the route for the development of high-efficiency, low-cost lead-free perovskite/organic TSCs in the future. Finally, more studies can be explored to extend the design of the proposed tandem by including new materials to serve as ETLs and HTLs in order to accomplish an appropriate design of the CBO and VBO, which is expected to boost the open circuit voltage and thereby the efficiency.

## Figures and Tables

**Table 1:** PV parameters of both experimental and simulated PSC and OSC structures under AM1.5G illumination.

	PSC		OSC	
	Experimental	Simulation	Experimental	Simulation
*J_sc_* (mA/cm^2^)	17.43	17.43	17.48	17.52
*V_oc_* (V)	0.600	0.606	0.741	0.738
FF (%)	72.0	72.36	NA	64.83
PCE (%)	7.50	7.64	NA	8.31

**Table 2:** Key tandem factors based on optical and full optoelectronic optimization.

Tandem PV Parameter	Optical Optimization	Optoelectronic Optimization
*J_sc_* (mA/cm^2^)	13.74	12.27
*V_oc_* (V)	1.486	1.498
FF (%)	65.24	76.16
PCE (%)	13.32	13.99

**Table 3:** Key tandem factors based on full optoelectronic optimization for 2-layer and 6-layer optimization under various situations of defects.

Optimization	Defects	*J_sc_* (mA/cm^2^)	*V_oc_* (V)	FF (%)	PCE (%)
2-layer	Initial	12.27	1.498	76.16	13.99
2-layer	Reduced in PSC	12.27	1.532	81.13	15.13
2-layer	Reduced in OSC	12.27	1.574	83.96	16.48
6-layer	Initial	13.29	1.548	77.64	15.96
6-layer	Reduced in PSC	13.29	1.548	77.69	16.00
6-layer	Reduced in OSC	13.29	1.580	82.15	17.46

## Data Availability

Not applicable.

## Supplementary Materials

The following supporting information can be downloaded at: https://www.mdpi.com/article/10.3390/polym15030784/s1, Table S1: Single junction PSC parameters; Table S2: Single junction OSC parameters; Table S3: The references used in obtaining the refractive index n and extinction coefficient k of the various materials of the tandem layers; Figure S1: Molecular structures of (a) DPPEZnP-TBO, (b) fullerene PC_61_BM.

## References

[1] Andreani L.C., Bozzola A., Kowalczewski P., Liscidini M., Redorici L. (2019). Silicon solar cells: Toward the efficiency limits. Adv. Phys..

[2] International Energy Agency (2019). Renewables 2019, Market Analysis and Forecast from 2019 to 2024. IEA Reports. https://www.iea.org/reports/renewables-2019.

[3] Yoshikawa K., Kawasaki H., Yoshida W., Irie T., Konishi K., Nakano K., Uto T., Adachi D., Kanematsu M., Uzu H. (2017). Silicon heterojunction solar cell with interdigitated back contacts for a photoconversion efficiency over 26%. Nat. Energy.

[4] Rühle S. (2016). Tabulated values of the Shockley–Queisser limit for single junction solar cells. Sol. Energy.

[5] Yang T.-C., Lee B.-S., Yen T.-J. (2012). Minimizing reflection losses from metallic electrodes and enhancing photovoltaic performance using the Si-micrograting solar cell with vertical sidewall electrodes. Appl. Phys. Lett..

[6] Zhang Y., Fan Z., Zhang W., Ma Q., Jiang Z., Ma D. (2018). High performance hybrid silicon micropillar solar cell based on light trapping characteristics of Cu nanoparticles. AIP Adv..

[7] Garnett E.C., Yang P. (2008). Silicon Nanowire Radial p−n Junction Solar Cells. J. Am. Chem. Soc..

[8] Salem M.S., Zekry A., Shaker A., Abouelatta M. Design and simulation of proposed low cost solar cell structures based on heavily doped silicon wafers. Proceedings of the 2016 IEEE 43rd Photovoltaic Specialists Conference (PVSC).

[9] Salem M.S., Zekry A., Shaker A., Abouelatta M., Abdolkader T.M. (2019). Performance enhancement of a proposed solar cell microstructure based on heavily doped silicon wafers. Semicond. Sci. Technol..

[10] Salem M.S., Alzahrani A.J., Ramadan R.A., Alanazi A., Shaker A., Abouelatta M., Gontrand C., Elbanna M., Zekry A. (2020). Physically Based Analytical Model of Heavily Doped Silicon Wafers Based Proposed Solar Cell Microstructure. IEEE Access.

[11] Salem M.S., Zekry A., Shaker A., Abouelatta M., Almurayziq T.S., Alshammari M.T., El-Banna M.M. (2022). Performance Improvement of npn Solar Cell Microstructure by TCAD Simulation: Role of Emitter Contact and ARC. Energies.

[12] Okil M., Salem M.S., Abdolkader T.M., Shaker A. (2021). From Crystalline to Low-cost Silicon-based Solar Cells: A Review. Silicon.

[13] (2021). UNIST, EPFL Claim 25.6% Efficiency World Record for Perovskite Solar Cell. PVMag. https://www.pv-magazine.com/2021/04/06/unist-epfl-claim-25-6-efficiency-world-record-for-perovskite-solar-cell/.

[14] Jacak J.E., Jacak W.A. (2022). Routes for Metallization of Perovskite Solar Cells. Materials.

[15] Zhu L., Zhang M., Xu J., Li C., Yan J., Zhou G., Zhong W., Hao T., Song J., Xue X. (2022). Single-junction organic solar cells with over 19% efficiency enabled by a refined double-fibril network morphology. Nat. Mater..

[16] Chen X., Jia Z., Chen Z., Jiang T., Bai L., Tao F., Chen J., Chen X., Liu T., Xu X. (2020). Efficient and Reproducible Monolithic Perovskite/Organic Tandem Solar Cells with Low-Loss Interconnecting Layers. Joule.

[17] Eperon G.E., Leijtens T., Bush K.A., Prasanna R., Green T., Wang J.T.-W., McMeekin D.P., Volonakis G., Milot R.L., May R. (2016). Perovskite-perovskite tandem photovoltaics with optimized band gaps. Science.

[18] Lin R., Xiao K., Qin Z., Han Q., Zhang C., Wei M., Saidaminov M.I., Gao Y., Xu J., Xiao M. (2019). Monolithic all-perovskite tandem solar cells with 24.8% efficiency exploiting comproportionation to suppress Sn(ii) oxidation in precursor ink. Nat. Energy.

[19] Zhao D., Chen C., Wang C., Junda M.M., Song Z., Grice C.R., Yu Y., Li C., Subedi B., Podraza N.J. (2018). Efficient two-terminal all-perovskite tandem solar cells enabled by high-quality low-bandgap absorber layers. Nat. Energy.

[20] Hou Y., Aydin E., De Bastiani M., Xiao C., Isikgor F.H., Xue D.-J., Chen B., Chen H., Bahrami B., Chowdhury A.H. (2020). Efficient tandem solar cells with solution-processed perovskite on textured crystalline silicon. Science.

[21] Green M.A., Dunlop E.D., Hohl-Ebinger J., Yoshita M., Kopidakis N., Hao X. (2020). Solar cell efficiency tables (version 56). Prog. Photovolt. Res. Appl..

[22] De Bastiani M., Babics M., Aydin E., Subbiah A.S., Xu L., De Wolf S. (2022). All Set for Efficient and Reliable Perovskite/Silicon Tandem Photovoltaic Modules?. Sol. RRL.

[23] Zeng Q., Liu L., Xiao Z., Liu F., Hua Y., Yuan Y., Ding L. (2019). A two-terminal all-inorganic perovskite/organic tandem solar cell. Sci. Bull..

[24] Xie S., Xia R., Chen Z., Tian J., Yan L., Ren M., Li Z., Zhang G., Xue Q., Yip H.-L. (2020). Efficient monolithic perovskite/organic tandem solar cells and their efficiency potential. Nano Energy.

[25] Liu L., Xiao Z., Zuo C., Ding L. (2021). Inorganic perovskite/organic tandem solar cells with efficiency over 20%. J. Semicond..

[26] Chen W., Zhu Y., Xiu J., Chen G., Liang H., Liu S., Xue H., Birgersson E., Ho J.W., Qin X. (2022). Monolithic perovskite/organic tandem solar cells with 23.6% efficiency enabled by reduced voltage losses and optimized interconnecting layer. Nat. Energy.

[27] Brinkmann K.O., Becker T., Zimmermann F., Kreusel C., Gahlmann T., Theisen M., Haeger T., Olthof S., Tückmantel C., Günster M. (2022). Perovskite–organic tandem solar cells with indium oxide interconnect. Nature.

[28] Alahmadi A.N.M. (2022). Design of an Efficient PTB7:PC70BM-Based Polymer Solar Cell for 8% Efficiency. Polymers.

[29] Moiz S.A., Alzahrani M.S., Alahmadi A.N.M. (2022). Electron Transport Layer Optimization for Efficient PTB7:PC_70_BM Bulk-Heterojunction. Solar Cells.

[30] Abdelaziz W., Zekry A., Shaker A., Abouelatta M. (2020). Numerical study of organic graded bulk heterojunction solar cell using SCAPS simulation. Sol. Energy.

[31] Madan J., Shivani, Pandey R., Sharma R. (2020). Device simulation of 17.3% efficient lead-free all-perovskite tandem solar cell. Sol. Energy.

[32] Duha A.U., Borunda M.F. (2022). Optimization of a Pb-free all-perovskite tandem solar cell with 30.85% efficiency. Opt. Mater..

[33] Pandey R., Sharma S., Madan J., Sharma R. (2021). Numerical simulations of 22% efficient all-perovskite tandem solar cell utilizing lead-free and low lead content halide perovskites. J. Micromech. Microeng..

[34] Abdelaziz S., Zekry A., Shaker A., Abouelatta M. (2022). Investigation of lead-free MASnI3-MASnIBr2 tandem solar cell: Numerical simulation. Opt. Mater..

[35] Hussain K., Gagliardi A.I. (2022). Modelling tandem/multi-junction hybrid perovskite—Organic solar cells: A combined drift—Diffusion and kinetic Monte Carlo study. Sol. Energy.

[36] Rossi D., Forberich K., Matteocci F., der Maur M.A., Egelhaaf H.-J., Brabec C.J., Di Carlo A. (2022). Design of Highly Efficient Semitransparent Perovskite/Organic Tandem Solar Cells. Sol. RRL.

[37] Chen M., Kapil G., Li Y., Kamarudin M.A., Baranwal A.K., Nishimura K., Sahamir S.R., Sanehira Y., Li H., Ding C. (2022). Large synergy effects of doping, a site substitution, and surface passivation in wide bandgap Pb-free ASnI2Br perovskite solar cells on efficiency and stability enhancement. J. Power Sources.

[38] Li M., Gao K., Wan X., Zhang Q., Kan B., Xia R., Liu F., Yang X., Feng H., Ni W. (2016). Solution-processed organic tandem solar cells with power conversion efficiencies > 12%. Nat. Photonics.

[39] Neukom M.T., Schiller A., Züfle S., Knapp E., Ávila J., Pérez-Del-Rey D., Dreessen C., Zanoni K.P., Sessolo M., Bolink H.J. (2019). Consistent Device Simulation Model Describing Perovskite Solar Cells in Steady-State, Transient, and Frequency Domain. ACS Appl. Mater. Interfaces.

[40] Ravishankar S., Liu Z., Rau U., Kirchartz T. (2022). Multilayer Capacitances: How Selective Contacts Affect Capacitance Measurements of Perovskite Solar Cells. PRX Energy.

[41] Gu X., Lai X., Zhang Y., Wang T., Tan W.L., McNeill C.R., Liu Q., Sonar P., He F., Li W. (2022). Organic Solar Cell With Efficiency Over 20% and V_OC_ Exceeding 2.1 V Enabled by Tandem with All-Inorganic Perovskite and Thermal Annealing-Free Process. Adv. Sci..

[42] Neukom M., Züfle S., Ruhstaller B. (2012). Reliable extraction of organic solar cell parameters by combining steady-state and transient techniques. Org. Electron..

[43] Alsaggaf S., Ashraf R.S., Purushothaman B., Chaturvedi N., McCulloch I., Laquai F., Khan J.I. (2021). Efficiency Limits in Wide-Bandgap Ge-Containing Donor Polymer:Nonfullerene Acceptor Bulk Heterojunction Solar Cells. Phys. Status Solidi (RRL)-Rapid Res. Lett..

[44] Balawi A.H., Kan Z., Gorenflot J., Guarracino P., Chaturvedi N., Privitera A., Liu S., Gao Y., Franco L., Beaujuge P. (2020). Quantification of Photophysical Processes in All-Polymer Bulk Heterojunction Solar Cells. Sol. RRL.

[45] Semiconducting Thin Film Optics Simulator (SETFOS) Version 5.2 by Fluxim AG, Switzerland. http://www.fluxim.com.

[46] Lanz T., Ruhstaller B., Battaglia C., Ballif C. (2011). Extended light scattering model incorporating coherence for thin-film silicon solar cells. J. Appl. Phys..

[47] Pope M., Swenberg C.E. (1999). Electronic Processes in Organic Crystals and Polymers.

[48] Neukom M. (2019). Comprehensive Characterization and Modelling of Operation Mechanisms in Third Generation Solar Cells.

[49] Altazin S., Kirsch C., Knapp E., Stous A., Ruhstaller B. (2018). Refined drift-diffusion model for the simulation of charge transport across layer interfaces in organic semiconductor devices. J. Appl. Phys..

[50] Coropceanu V., Cornil J., da Silva Filho D.A., Olivier Y., Silbey R., Brédas J.L. (2007). Charge Transport in Organic Semiconductors. Chem. Rev..

[51] Coropceanu V., Cornil J. (2022). Analysis and optimization of organic tandem solar cells by full opto-electronic simulation. Front. Photonics.

[52] Qirong Z., Bao Z., Yongmao H., Liang L., Zhuoqi D., Zaixin X., Xiaobo Y. (2022). A study on numerical simulation optimization of perovskite solar cell based on CuI and C60. Mater. Res. Express.

[53] Stübinger T. (2005). Optische Modellierung und Charakterisierung von Organischen Donor-Akzeptor Solarzellen.

[54] Xiao L., Liang T., Gao K., Lai T., Chen X., Liu F., Russell T.P., Huang F., Peng X., Cao Y. (2017). Ternary Solar Cells Based on Two Small Molecule Donors with Same Conjugated Backbone: The Role of Good Miscibility and Hole Relay Process. ACS Appl. Mater. Interfaces.

[55] He Z., Zhong C., Su S., Xu M., Wu H., Cao Y. (2012). Enhanced power-conversion efficiency in polymer solar cells using an inverted device structure. Nat. Photonics.

[56] Ho C.H.Y., Kothari J., Fu X. (2021). and So, F. Interconnecting layers for tandem organic solar cells. Materials. Today Energy.

[57] Gu S., Lin R., Han Q., Gao Y., Tan H., Zhu J. (2020). Tin and Mixed Lead–Tin Halide Perovskite Solar Cells: Progress and their Application in Tandem Solar Cells. Adv. Mater..

