# Supplementary material for: Full Optoelectronic Simulation of Lead-Free Perovskite/Organic Tandem Solar Cells
Source: Polymers (Basel). 2023 Feb 3;15(3):784. doi: 10.3390/polym15030784 (PMC9918906; doi:10.3390/polym15030784)
Supplement: Supplementary file 1 [file polymers-15-00784-s001.zip › polymers-2131795-supplementary.pdf]

Supplementary Materials

Full Optoelectronic Simulation of lead-free Perovskite/Organic Tandem Solar cells

Marwa S. Salem, Ahmed Shaker, M. Abouelatta and Ahmed Saeed

ELECTRICAL MATERIAL PARAMETERS

Table S1. Single junction PSC parameters [S1, S2, S3]

	**ITO**	**PEDOT:PSS**	**Lead-free Perovskite***	**C60**	**Ag**
Thickness (nm)	150	31	212	25	130
HOMO (eV)		4.9	5.68	5.9	
LUMO (eV)		3.6	4.06	4.2	
ε_r		3.5	7	5	
N_c (cm^{-3})		10^{21}	2×10^{18}	2.2×10^{18}	
N_v (cm^{-3})		10^{21}	2×10^{18}	1.8×10^{19}	
N_A (cm^{-3})		10^{18}			
N_D (cm^{-3})				2×10^{18}	
μ_n (cm^2/Vs)		8×10^{-5}	0.01	0.08	
μ_p (cm^2/Vs)		8×10^{-5}	0.01	0.0035	
N_t (cm^{-3})			10^{13}		
E_t (eV)			0.9		
C_n (cm^{-3}s^{-1})			10^{-10}		
C_p (cm^{-3}s^{-1})			10^{-10}		
Work function (eV)	4.3				4.3

* The lead-free perovskite is $GA_{0.06}(FA_{0.8}Cs_{0.2})_{0.94}SnI_2Br$. At A site, a bigger organic cation, guanidinium (GA+) is used in addition to formamidinium (FA), and cesium (Cs) cations. At the B-site, bivalent cation tin (Sn) is placed. X is a combination of monovalent halide anions I and Br. (Remark: the perovskite material structure is generally denoted by ABX_3 where A is a monovalent cation, B is a divalent cation, and X is a halide anion.)

Table S2. Single junction OSC parameters [S1, S4]

	**ITO**	**PEDOT:PSS**	**DPPEZnP-TBO: PC61BM**	**PFN**	**Al**
Thickness (nm)	116	15	117.5	5	70
HOMO (eV)		4.9	5.23	7	
LUMO (eV)		3.6	3.9	3.9	
ε_r		3.5	3.5	3.5	
N_c (cm^{-3})		10^{21}	10^{21}	10^{21}	
N_v (cm^{-3})		10^{21}	10^{21}	10^{21}	
N_A (cm^{-3})		10^{18}			
N_D (cm^{-3})					
μ_n (cm^2/Vs)		8×10^{-5}	5×10^{-4}	10^{-4}	
μ_p (cm^2/Vs)		8×10^{-5}	5×10^{-4}	10^{-6}	
N_t (cm^{-3})			2×10^{14}		
E_t (eV)			0.5		
C_n (cm^{-3}s^{-1})			10^{-10}		
C_p (cm^{-3}s^{-1})			10^{-10}		
Work function (eV)	4.4				4.1

OPTICAL MATERIAL DATA

Table S3. The references used in obtaining the refractive index n and extinction coefficient k of the various materials of the tandem layers

Subcell	Layer	REF
PSC	ITO	Setfos material database (from Woollam VASE)
	PEDOT:PSS	Setfos material database (from www.hcstarck.com)
	$ASnI_2Br$ lead-free perovskite	[S3]
	C60	Setfos material database (Measured by Thomas Stuebinger at the University of Bayreuth [S5])
OSC	PEDOT:PSS	Setfos material database (from www.hcstarck.com)
	DPPEZnP-TBO:PC61BM	[S6]
	PFN	[S7]
	Al	Setfos material database (from Woollam)

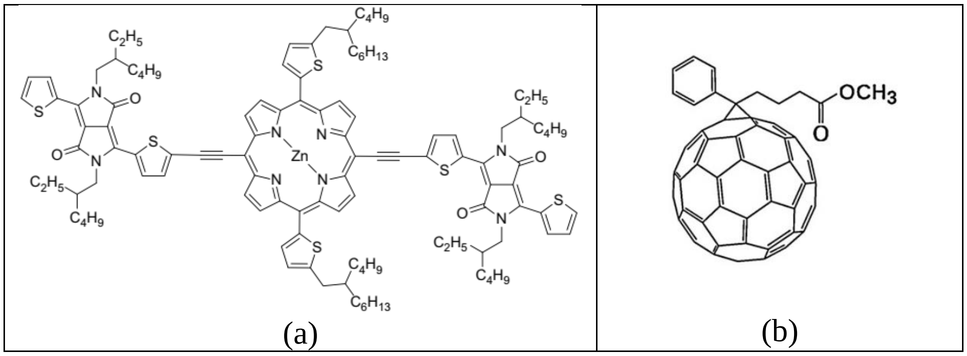

Figure S1 Molecular structures of (a) DPPEZnP-TBO, (b) fullerene $PC_{61}BM$.

References

[S1] Coropceanu, V.; Cornil, J. Analysis and optimization of organic tandem solar cells by full opto-electronic simulation. *Frontiers in Photonics* **2022**, *3*, 16.

[S2] Qirong, Z.; Bao, Z.; Yongmao, H.; Liang, L.; Zhuoqi, D.; Zaixin, X.; Xiaobo, Y. A study on numerical simulation optimization of perovskite solar cell based on CuI and C60. *Materials Research Express* **2022**, *9(3)*, 036401, DOI 10.1088/2053-1591/ac5a34.

[S3] Chen, M.; Kapil, G.; Li, Y.; Kamarudin, M. A.; Baranwal, A. K.; Nishimura, K.; Hayase, S. Large synergy effects of doping, a site substitution, and surface passivation in wide bandgap Pb-free $ASnI_2Br$ perovskite solar cells on efficiency and stability enhancement. *Journal of Power Sources* **2022**, *520*, 230848, https://doi.org/10.1016/j.jpowsour.2021.230848.

[S4] C. Li, M.; Gao, K.; Wan, X.; Zhang, Q.; Kan, B.; Xia, R.; Chen, Y. Solution-processed organic tandem solar cells with power conversion efficiencies> 12%. *Nature Photonics* **2017**, *11(2)*, 85-90, DOI: 10.1038/NPHOTON.2016.240.

[S5] Stübinger, T. Optische modellierung und charakterisierung von organischen donor-akzeptor solarzellen. *Perspektivenverl.* Ph.D. Thesis, **2005**.

[S6] Xiao, L.; Liang, T.; Gao, K.; Lai, T.; Chen, X.; Liu, F.; Cao, Y. Ternary solar cells based on two small molecule donors with same conjugated backbone: the role of good miscibility and hole relay process. *ACS Applied Materials & Interfaces* **2017**, *9(35)*, 29917-29923, https://doi.org/10.1021/acsami.7b07960

[S7] He, Z.; Zhong, C.; Su, S.; Xu, M.; Wu, H.; Cao, Y. Enhanced power-conversion efficiency in polymer solar cells using an inverted device structure. *Nat. Photonics* **2012,** *6*, 591–595, doi:10.1038/nphoton.2012.190.
